# Supplementary figures and images for: Dynamic localization of SPO11-1 and conformational changes of meiotic axial elements during recombination initiation of maize meiosis
Source: PLoS Genet. 2020 Apr 20;16(4):e1007881. doi: 10.1371/journal.pgen.1007881 (PMC7192515; doi:10.1371/journal.pgen.1007881)

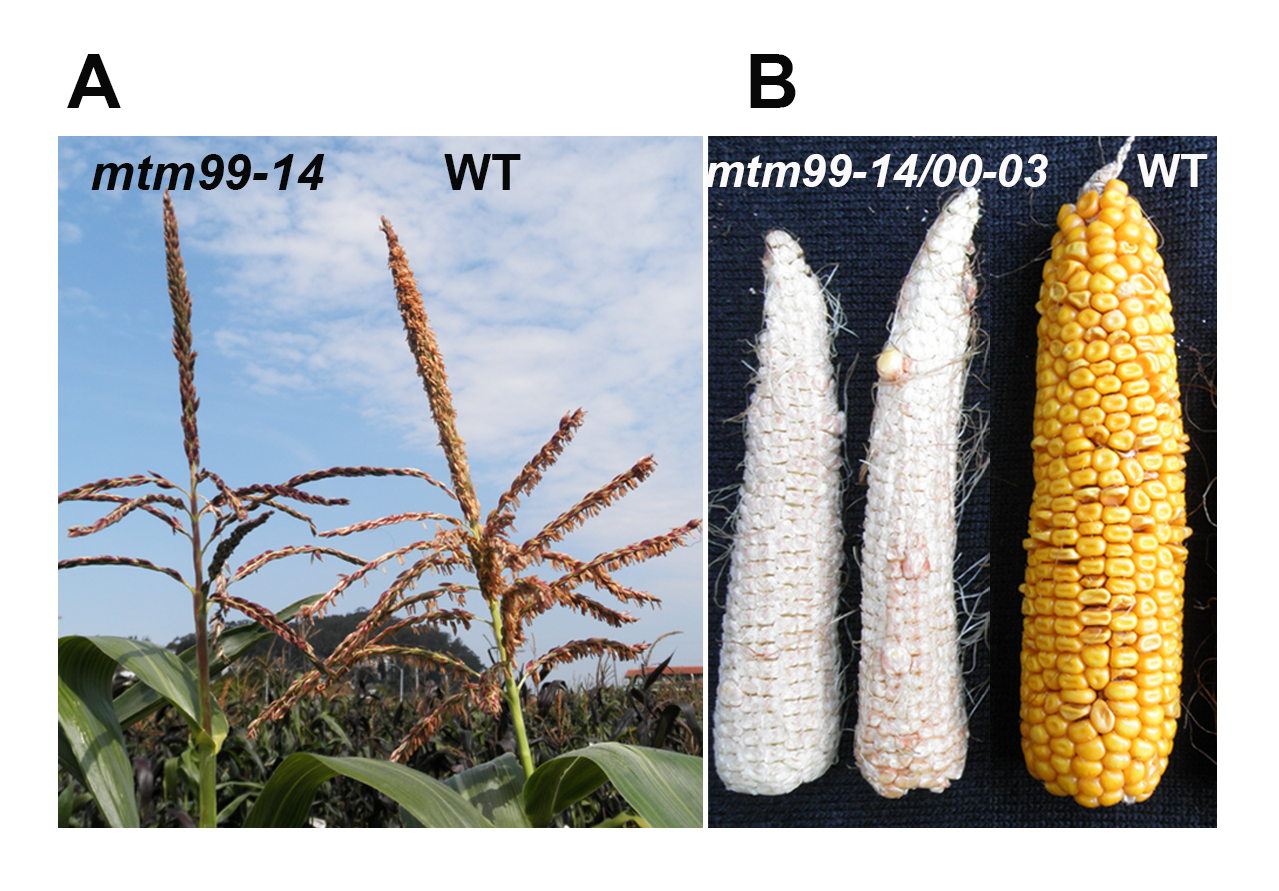

Supplement: S1 Fig — (A) Male inflorescence (tassel) of the mtm99-14 mutant and wild type (WT) at time of pollen shedding. The WT plant exhibits protruding anthers and shed pollen. Protruding anthers are very rare in mutant plants and they do not shed pollen grains. (B) The two maize ears on the left are from heteroallelic mtm99-14/mtm00-03 plants pollinated with WT (Mo17) pollen and mainly present shriveled ovules with few kernels. The ear on the right is from a WT sibling plant that is full of kernels. (TIF) [file pgen.1007881.s001.tif]

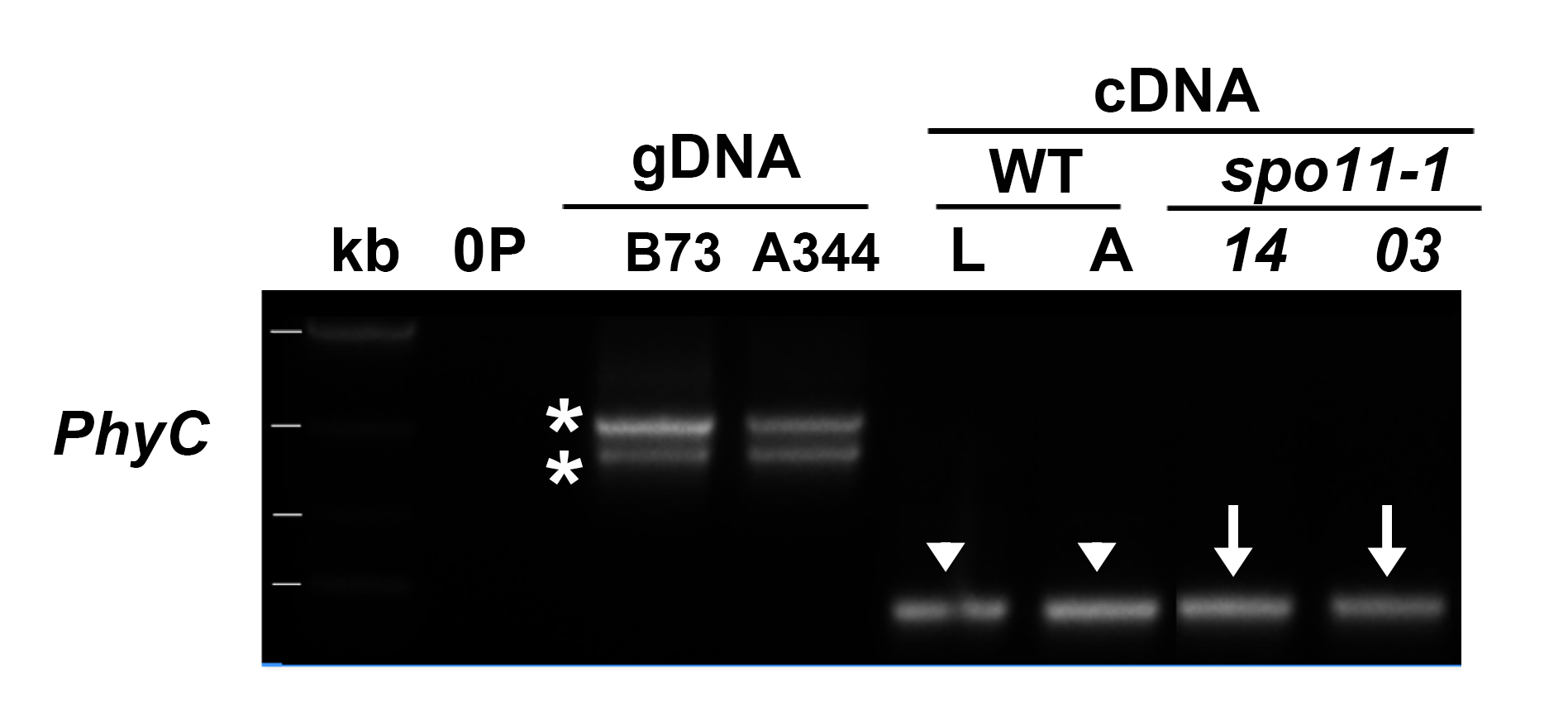

Supplement: S2 Fig — Given 94% identity between the PhyC1 and PhyC2 coding regions, both PhyC1 and PhyC2 fragments (asterisks) were amplified from genomic DNA (gDNA) of B73 and A344 WT using primers AR105/AR106 (S7 Table). 0P represents a negative PCR control lacking DNA template. By RT-PCR, the same primers amplified both transcripts (arrowheads) of identical size from WT leaf (L) and anther (A). However, sequencing results confirmed that only the PhyC1 transcript (arrows) was detected in the mtm99-14 (14) and mtm00-03 (03) mutants. (TIF) [file pgen.1007881.s002.tif]

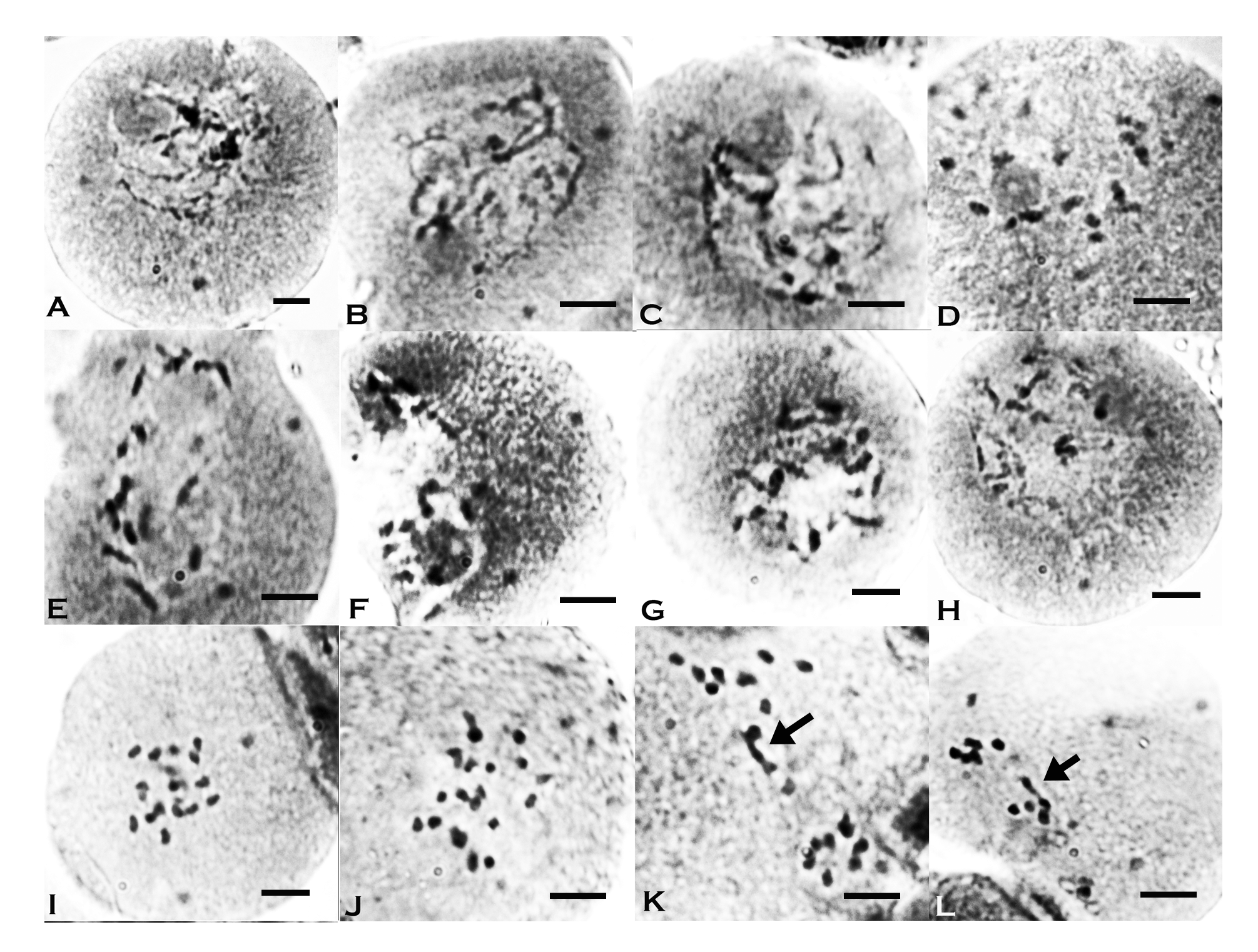

Supplement: S3 Fig — (A) spo11-1-1 meiocyte at pachytene. (B-C) spo11-1-1 meiocytes at diplotene. (D-H) spo11-1-1 meiocytes at diakinesis mostly exhibit univalents.(I-L) spo11-1-1 meiocytes at metaphase I mostly exhibit univalents and occasional bivalents (arrows in K and L). Scale bar represents 10 μm. (TIF) [file pgen.1007881.s003.tif]

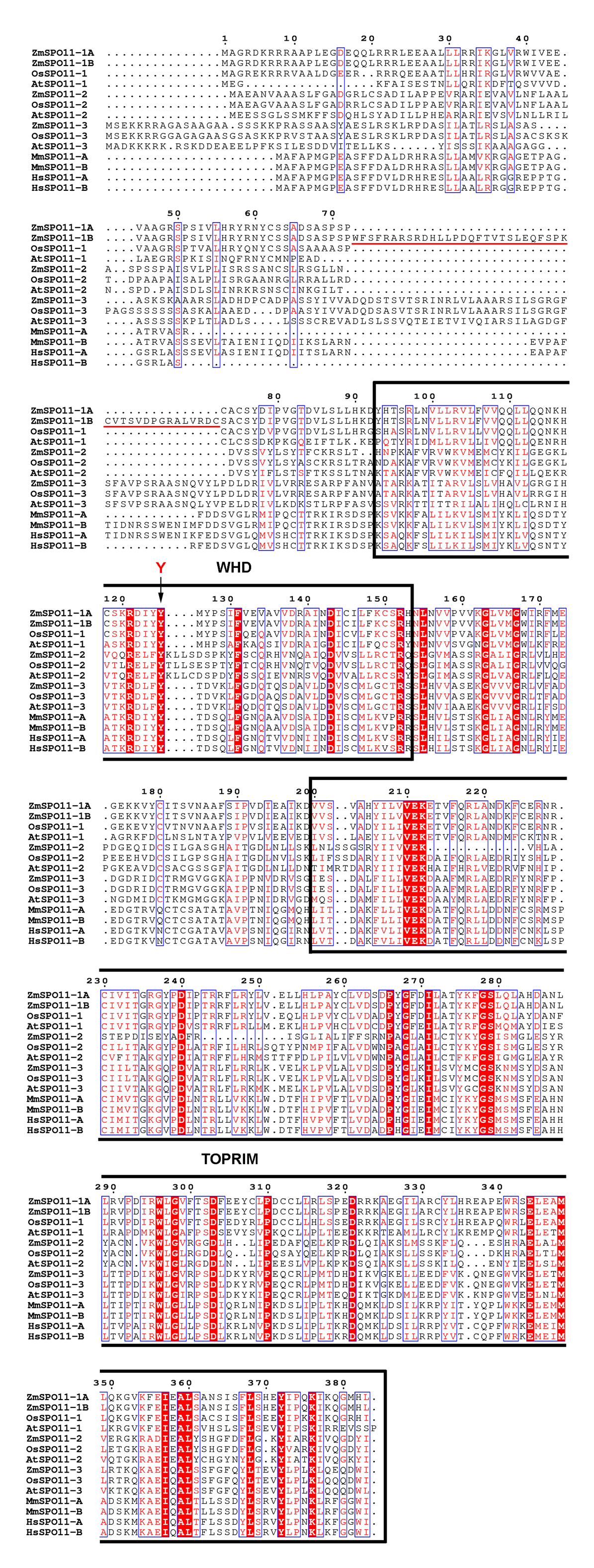

Supplement: S4 Fig — Conserved residues are highlighted in red. The conserved tyrosines (Y) in the WHD and TOPRIM domains are indicated. The additional 43-amino acid domain in SPO11-1β is underlined (red), which exhibits positional similarity to regions of SPO11-3 and the mammalian SPO11-β isoform. (TIF) [file pgen.1007881.s004.tif]

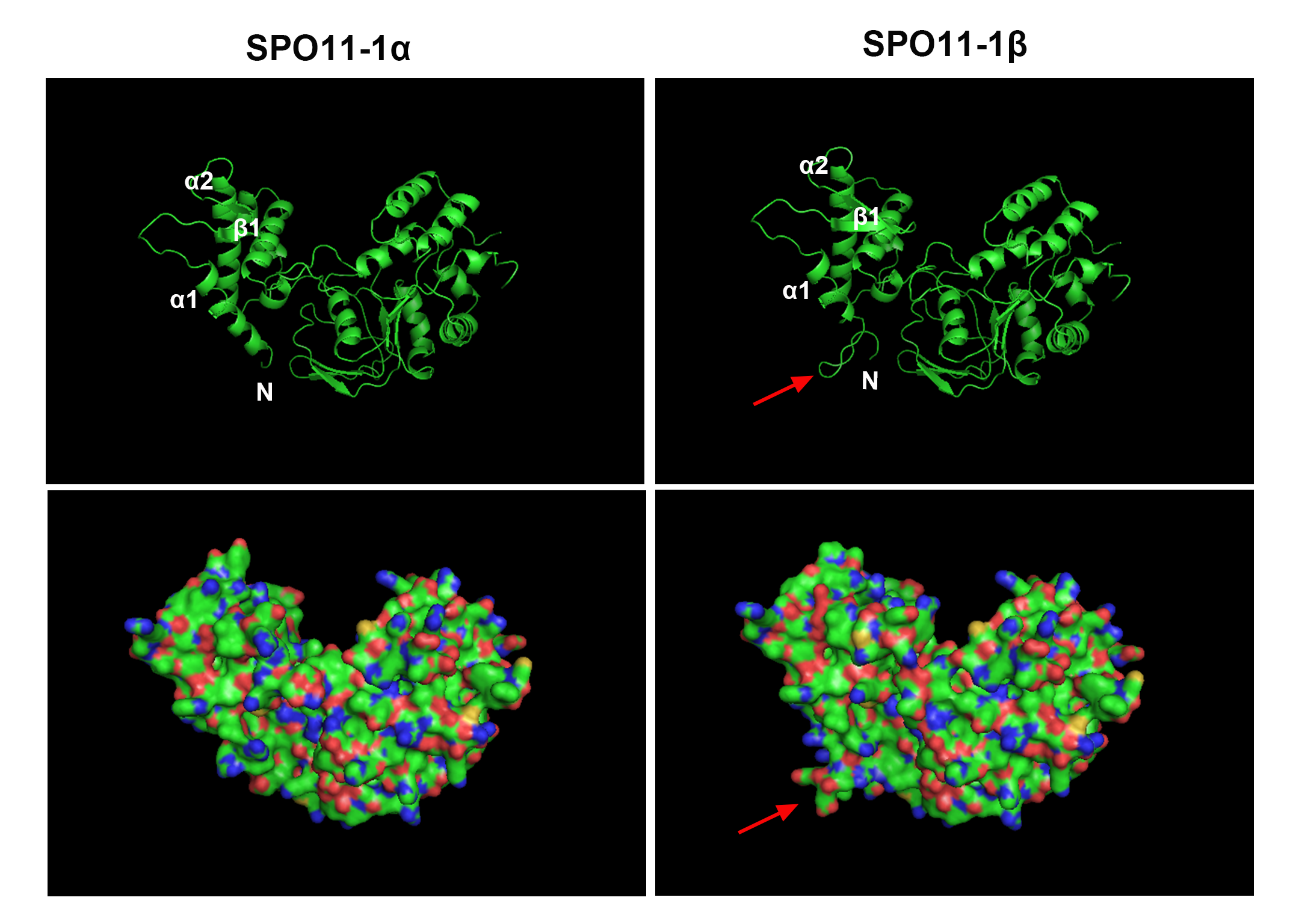

Supplement: S5 Fig — Predicted structures were obtained using Phyre2 and visualized using the PyMOL ‘cartoon’ (top) and ‘surface’ (bottom) tools. The SPO11-1 structure is based on the defined crystal structure of the TOPVIA protein of Methanococcus jannaschii (PDB model: c2zbkA). It forms a horseshoe shape that can dimerize into a ring. The additional domain of 43 amino acids in SPO11-1β manifests as a protruding alpha-helical region (arrows) opposite the groove containing the DNA binding region and the tyrosine catalytic site. N represents the N-terminal. (TIF) [file pgen.1007881.s005.tif]

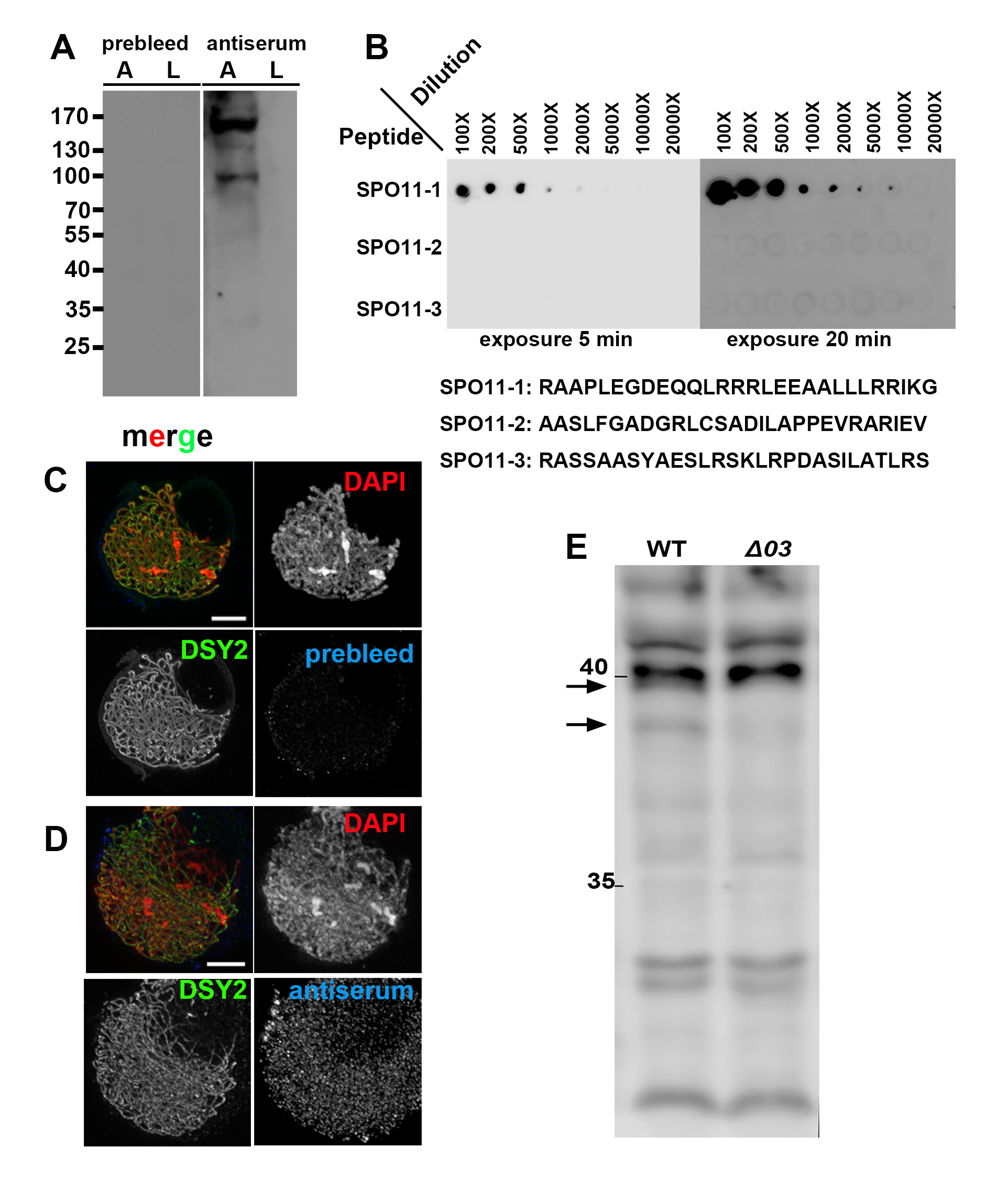

Supplement: S7 Fig — (A) Western blot analyses with rabbit pre-immune serum (prebleed) and anti-immune serum (antiserum) were used to determine background levels before immunization and to detect any generated IgG that may recognize target proteins from total protein extracts of anther (A) and leaf (L) tissues. (B) Maize SPO11-1, SPO11-2 and SPO11-3 proteins share some similarities (S4 Fig). To validate our SPO11-1 antibody specificity, dot blot analyses were performed using SPO11-1 antiserum (1:1000 dilution) against synthetic peptides of SPO11-1 antigen, SPO11-2 and SPO11-3 in corresponding regions. Their amino-acid sequences are listed below the dot blots. Serial dilutions of equal amounts (1 μg) of peptide were dotted for detection and blots were imaged using the UVP Biospectrum 600 system with exposure times of 5 or 20 min. (C-D) Pre-immune serum (C, prebleed) and anti-immune serum (D) were used to test SPO11-1 antibody in WT meiocytes at early zygotene stage by means of immunofluorescence analysis. (E) Western blot analysis using the affinity-purified SPO11-1 antibody for detection of potential SPO11-1 proteins in total protein extracts of WT and spo11-1-Δ03 (Δ03) anthers. Two weak bands (arrows) of ~40 kDa were detected in WT, but these were absent from the mutant sample. (TIF) [file pgen.1007881.s007.tif]

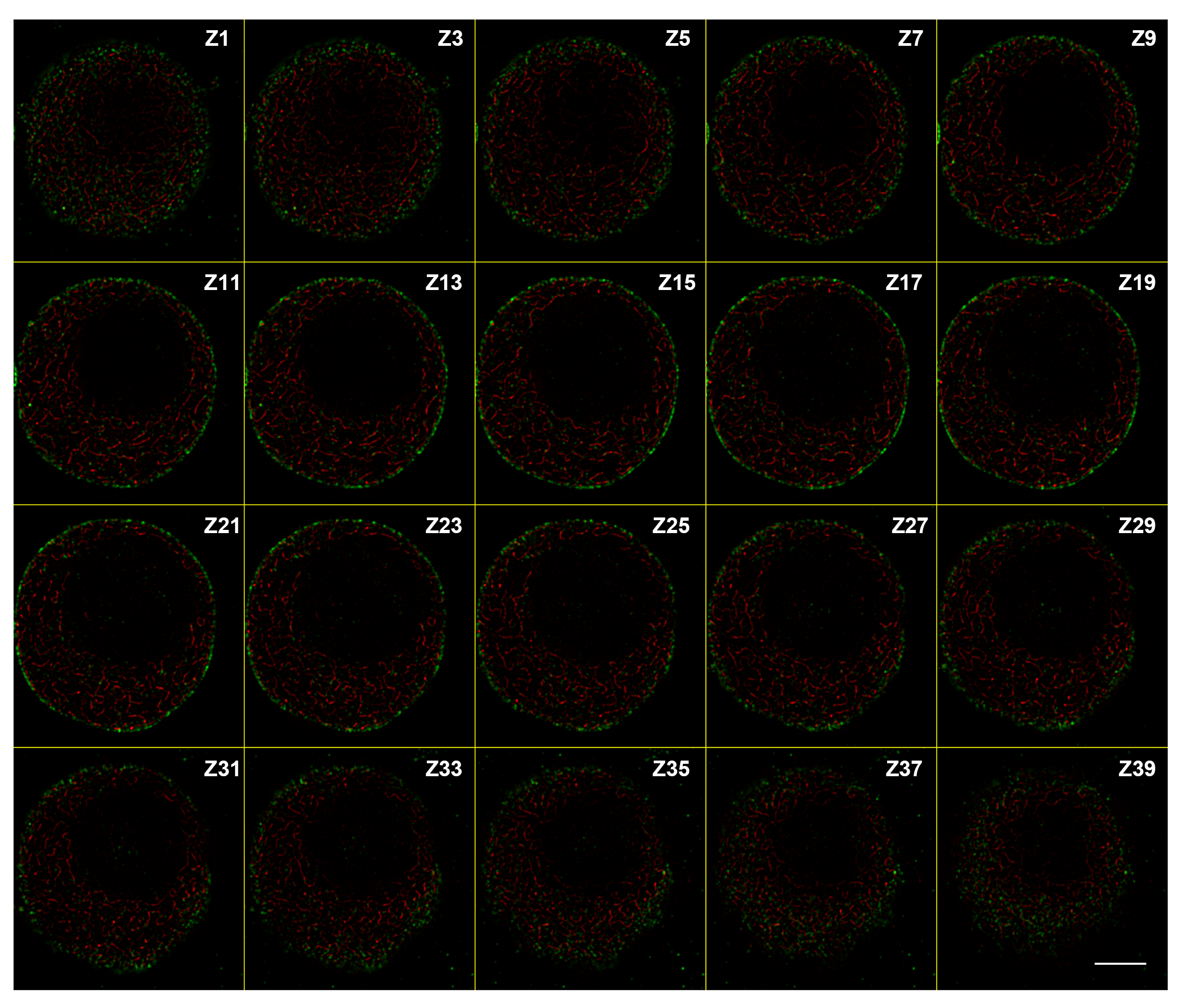

Supplement: S8 Fig — A montage of single Z sections with one Z-section intervals showing SPO11-1 (green) and DSY2 (red) signals. Scale bar represents 5 μm. (TIF) [file pgen.1007881.s008.tif]

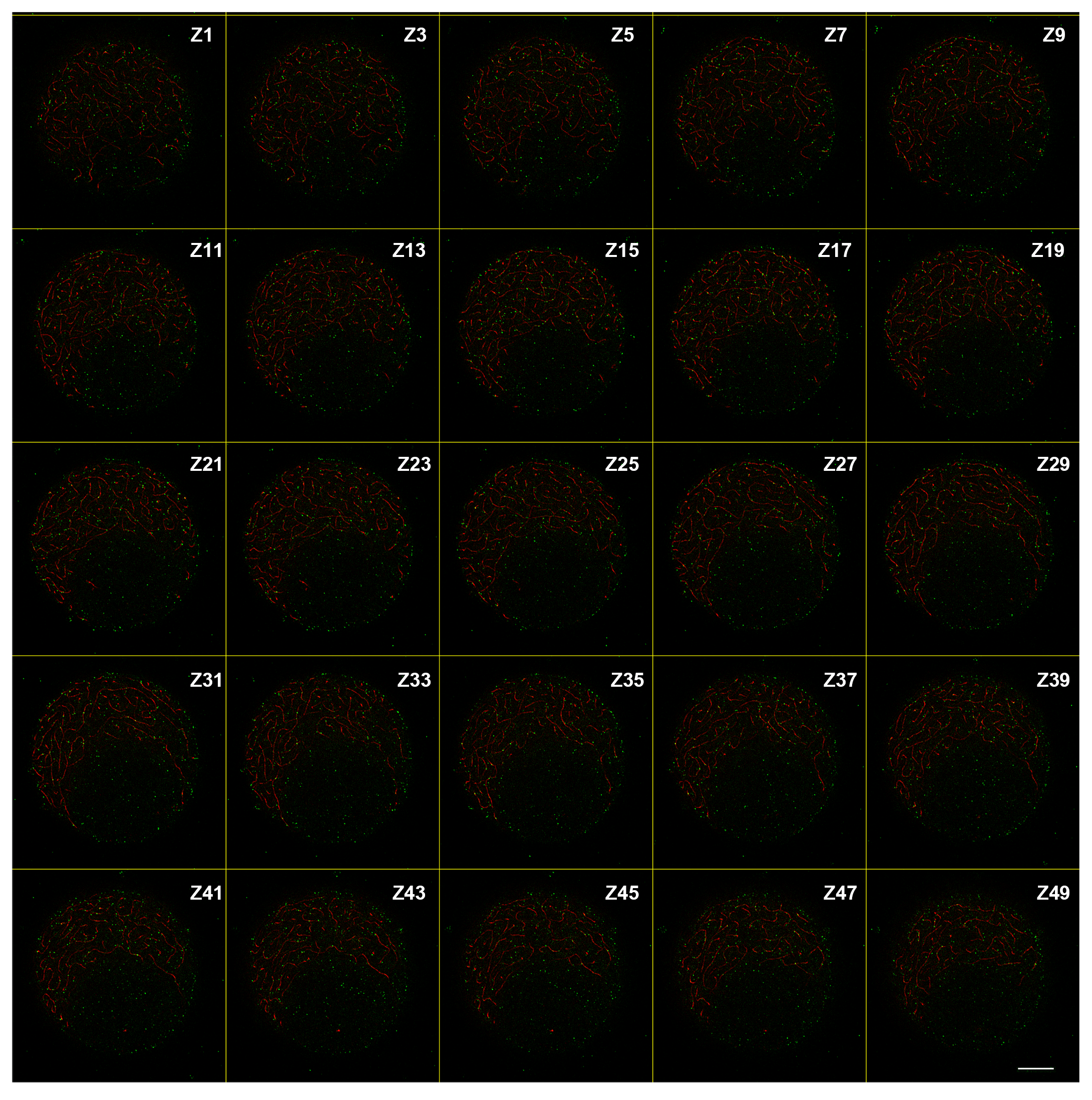

Supplement: S9 Fig — A montage of single Z sections with one Z-section intervals showing SPO11-1 (green) and DSY2 (red) signals. Scale bar represents 5 μm. (TIF) [file pgen.1007881.s009.tif]

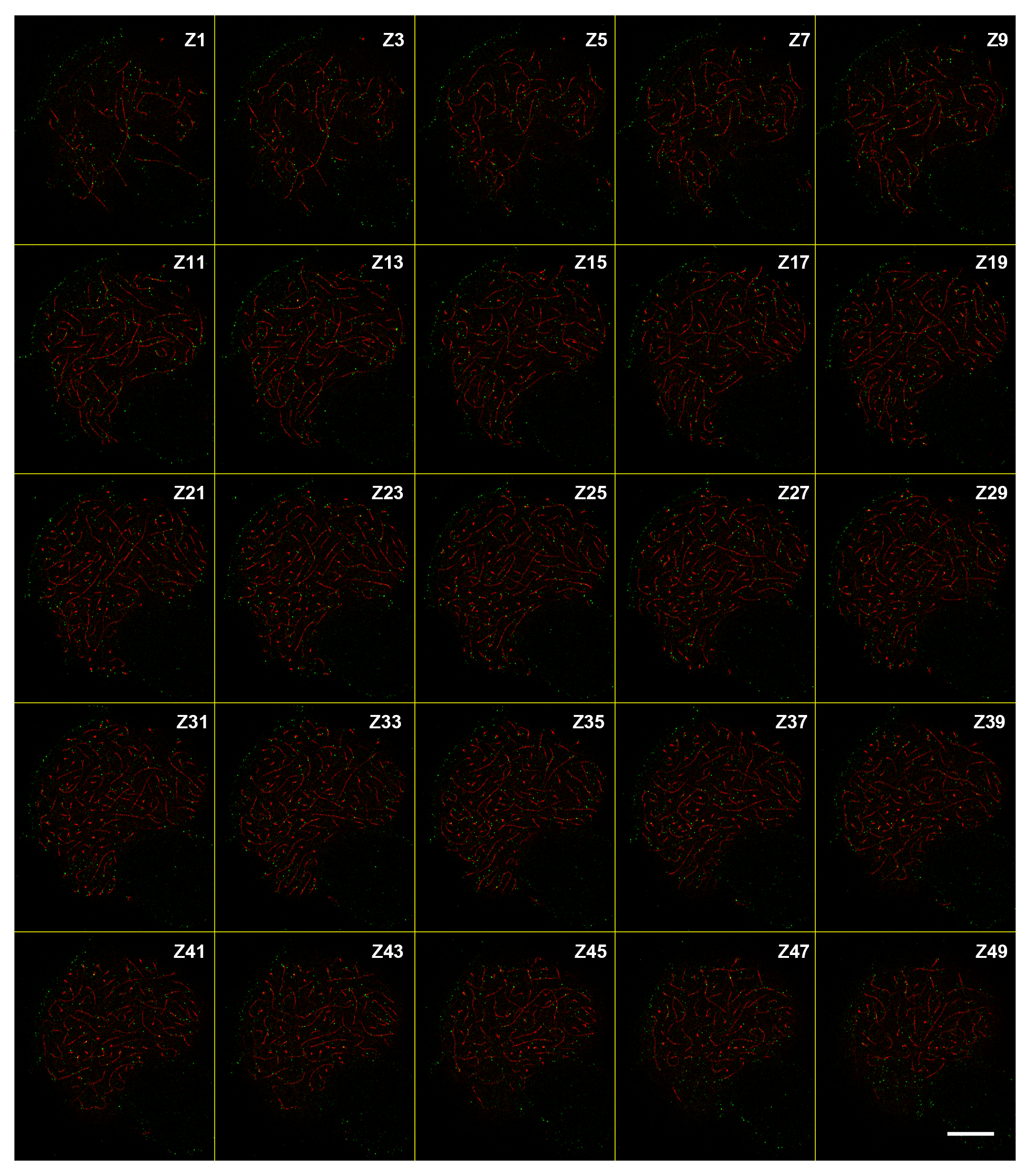

Supplement: S10 Fig — A montage of single Z sections with one Z-section intervals showing SPO11-1 (green) and DSY2 (red) signals. Note that Z11-Z19 exhibit pre-aligned and synapsing regions. Scale bar represents 5 μm. (TIF) [file pgen.1007881.s010.tif]

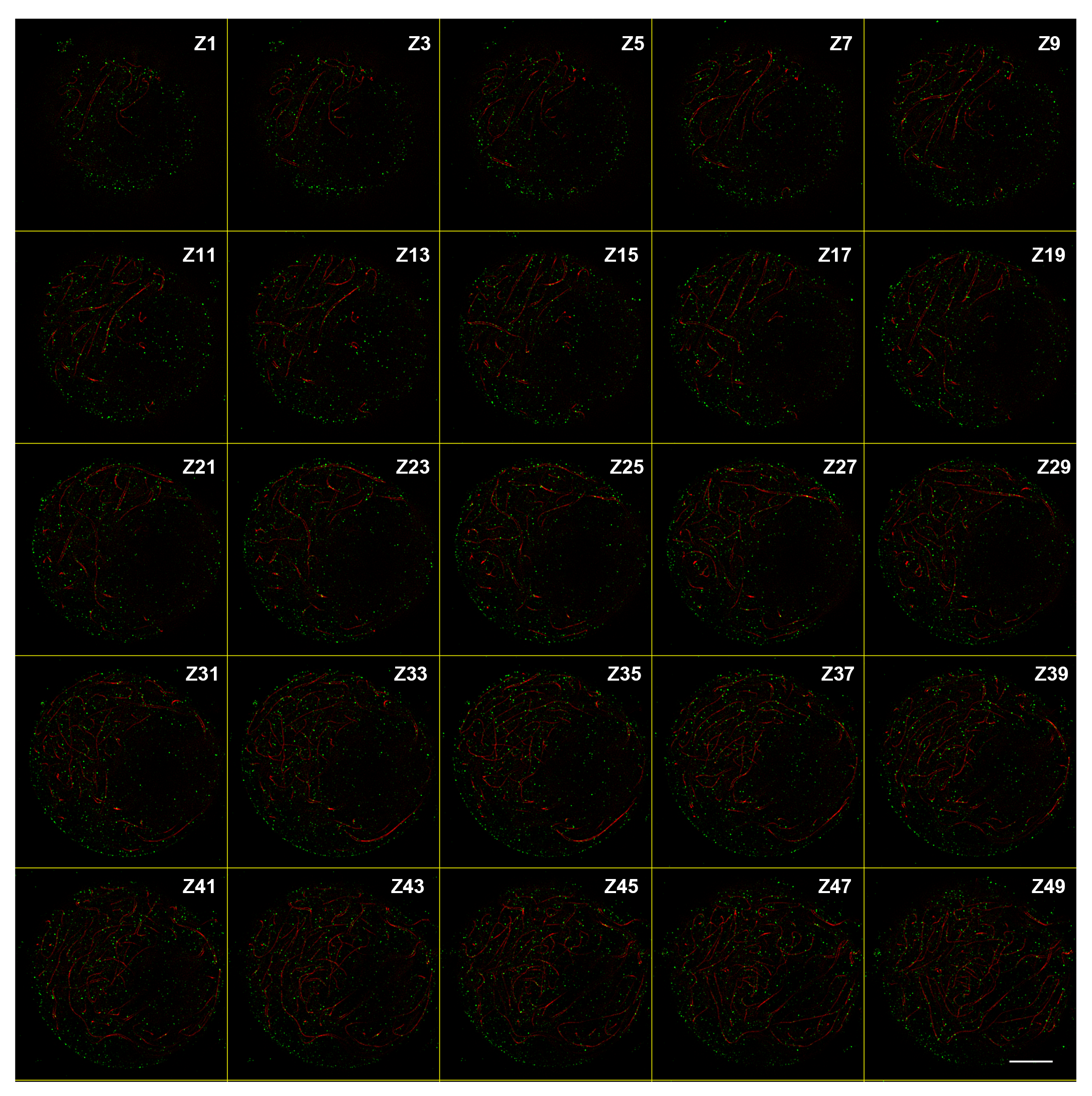

Supplement: S11 Fig — A montage of single Z sections with one Z-section intervals showing SPO11-1 (green) and DSY2 (red) signals. Scale bar represents 5 μm. (TIF) [file pgen.1007881.s011.tif]

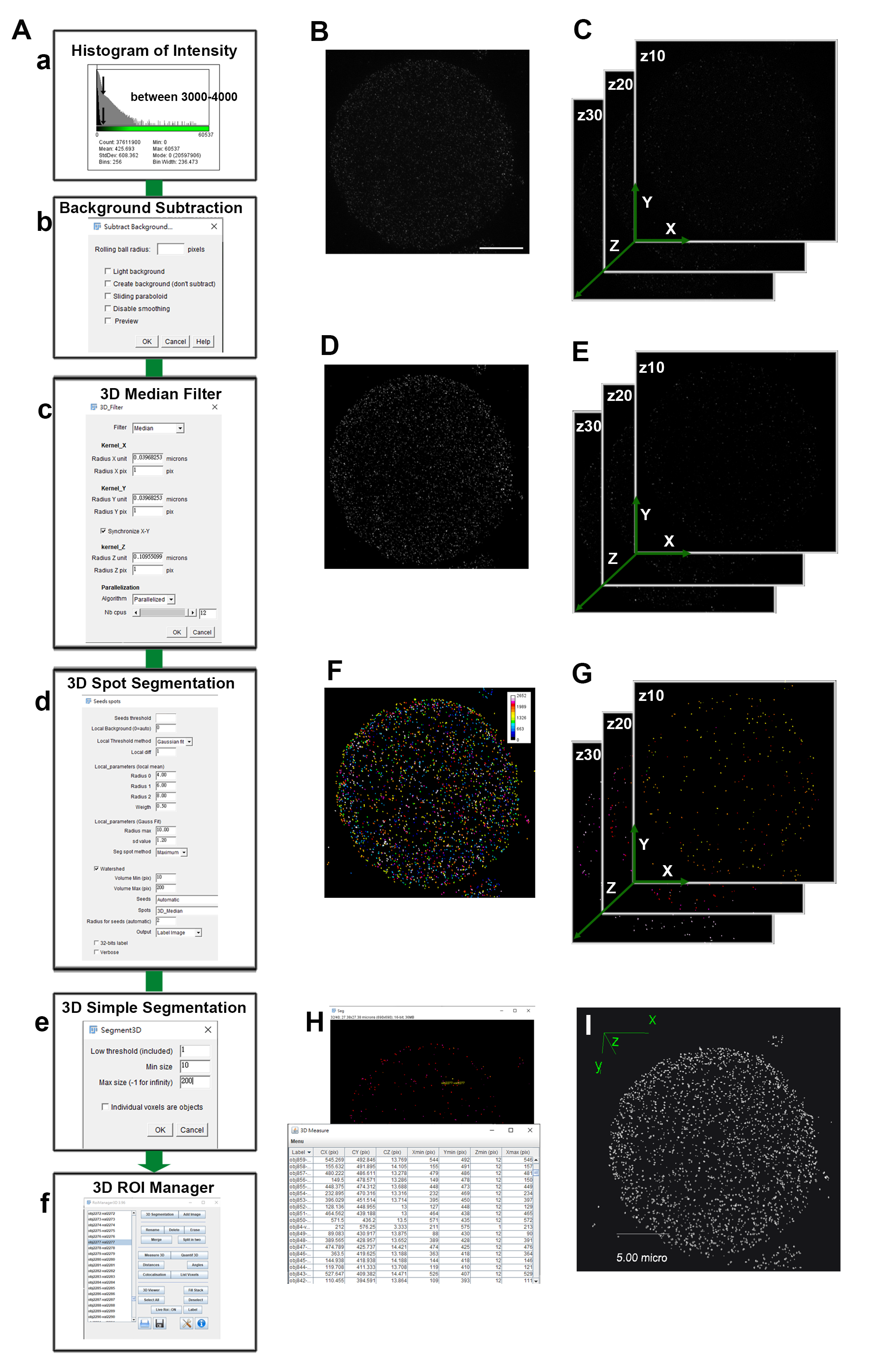

Supplement: S12 Fig — (A) Schematic workflow for segmentation and quantification of SPO11-1 signals. (a) Histogram of fluorescence intensity for all image stacks of a representative nucleus shown in B-C. The X-axis represents intensity values and the Y-axis shows the number of pixels found for each value. The black area displays a unimodal histogram, showing that the peak contains mostly lower intensity background, and the gray overlay shows a log-scaled version. The histogram of intensity is used to determine threshold values (arrows) that are usually between 3000–4000 for a 16-bit image. (b-c) Pre-processing step to enhance object contrast by rolling ball background subtraction and 3D median filtering. The resulting images are shown in D-E. (d) Detection and segmentation of SPO11-1 signals by 3D spot segmentation. using a threshold value defined by the histogram distribution in (a). This algorithm defines seeds of spots in original images using a threshold value defined by the histogram distribution in (a) and computed neighboring voxels as belonging to the object with criteria of volume of objects and local contrast (Gaussian fit). (e-f) Labeling of segmented objects by 3D simple segmentation and examination using 3D ROI manager. Geometric measurement of objects (H) was obtained using the Measure 3D tool. (B-C) Representative raw images of SPO11-1 signals shown in the maximal projection (B) and single Z sections (C). Scale bar represents 5 μm.(D-E) Pre-processed images of SPO11-1 signals shown in the maximal projection (D) and single Z sections (E).(F-G) Segmented objects shown in the maximal projection (F) and single Z sections (G). A total of 2652 3D objects was identified in the representative meiocyte. The color code represents numbered objects. (H) Labeled objects were inspected and measured using 3D ROI manager.(I) A surface-rendered image of SPO11-1 signals in the same nucleus visualized using 3D viewer. (TIF) [file pgen.1007881.s012.tif]

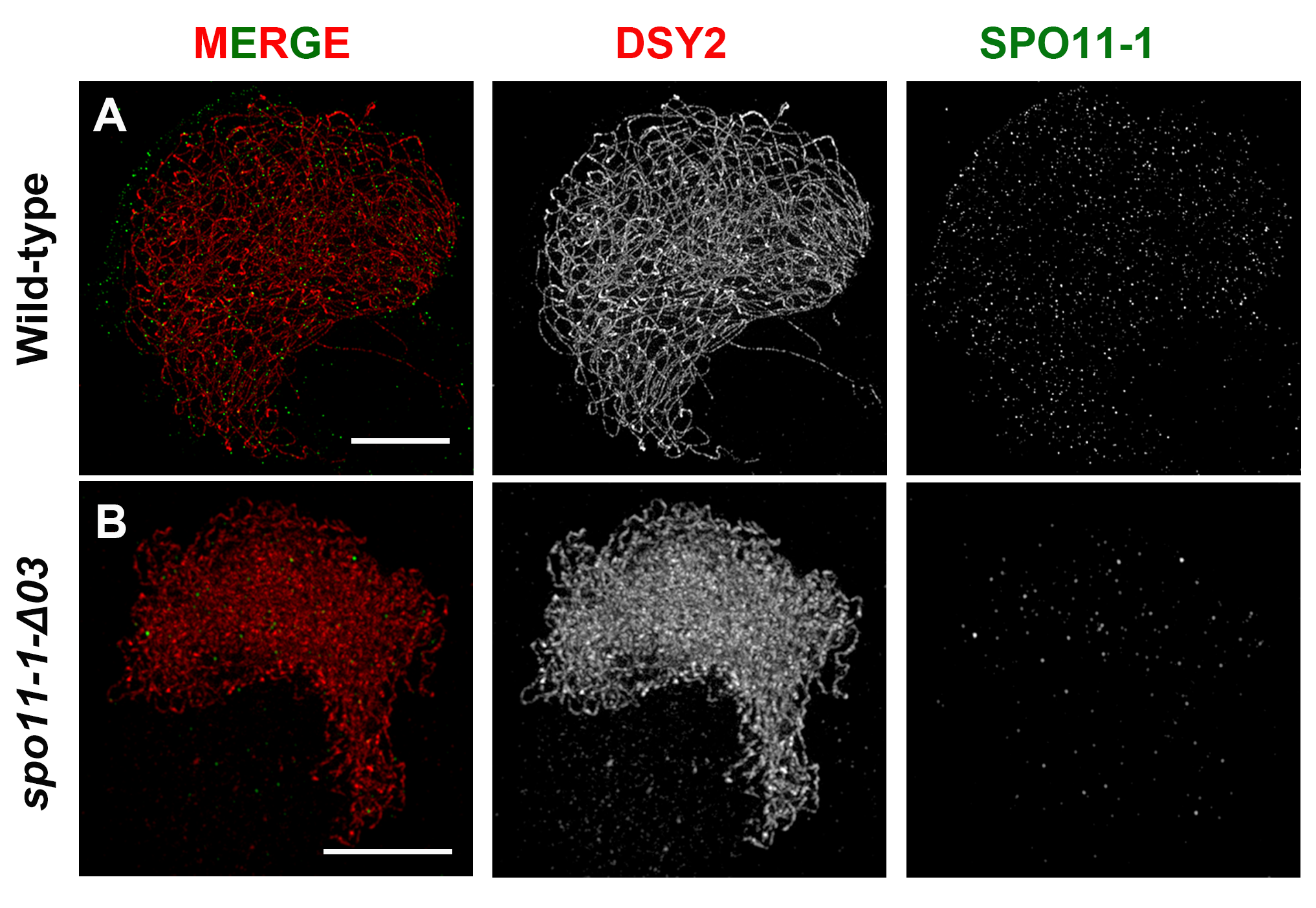

Supplement: S13 Fig — (A) A WT meiocyte showing chromosome axes labeled by DSY2 (red or gray) and SPO11-1 signals (green or gray).(B) A representative spo11-1-Δ03 meiocyte showing chromosome axes labeled by DSY2 (red or gray) and a few foci (green or gray) detected using a SPO11-1 antibody.Scale bar represents 5 μm. (TIF) [file pgen.1007881.s013.tif]

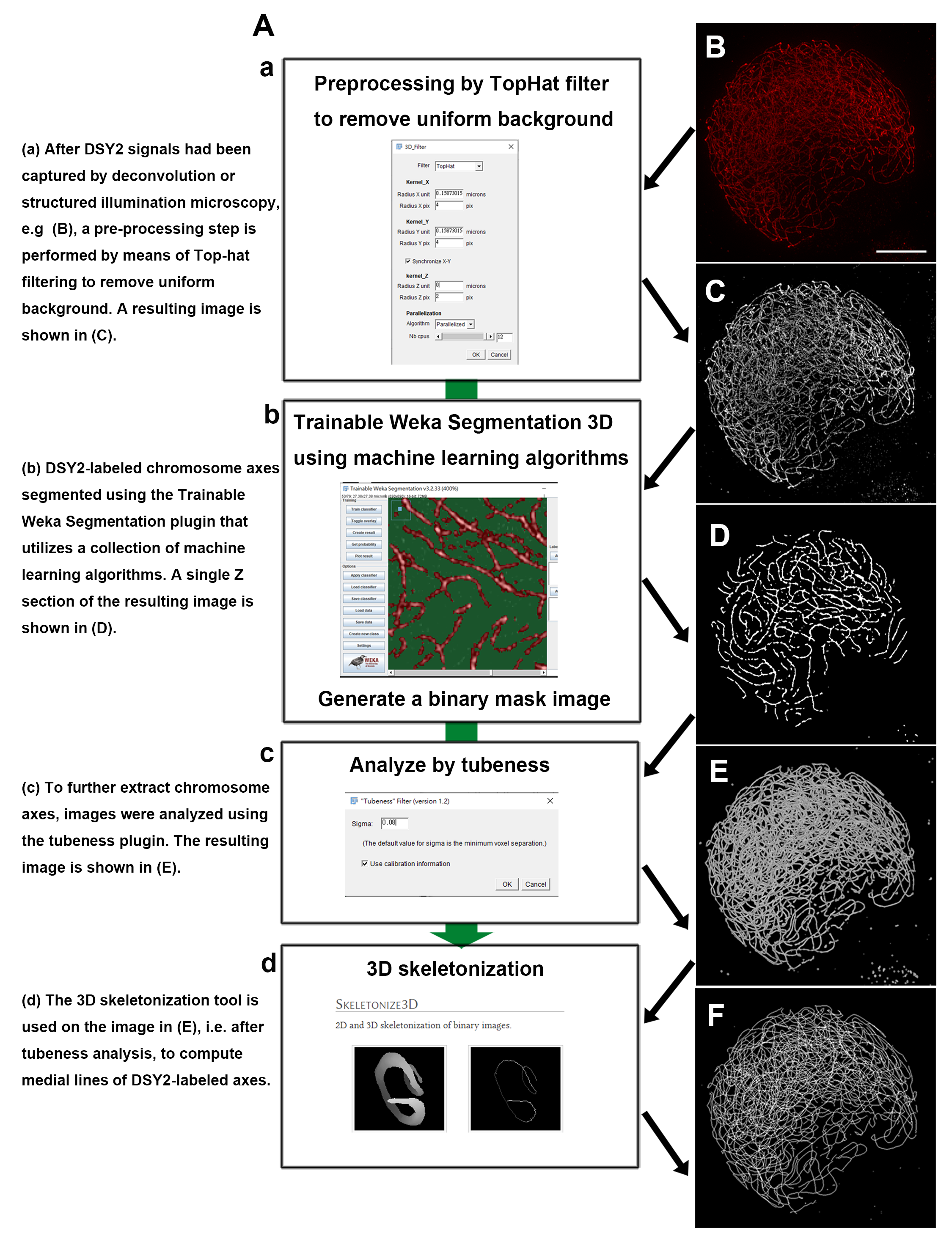

Supplement: S14 Fig — (A) Schematic workflow for segmentation of DSY2 signals. (a) After DSY2 signals had been captured by deconvolution microscopy or structured illumination microscopy (B), a pre-processing step is performed by means of Top-hat filtering to remove uniform background. A resulting image is shown in (C). (b) DSY2-labeled chromosome axes segmented using the Trainable Weka Segmentation plugin that utilizes a collection of machine learning algorithms. A single Z section of the resulting image is shown in (D). (c) To further extract chromosome axes, images were analyzed using the tubeness plugin. The resulting image is shown in (E). (d) The 3D skeletonization tool is used on the image in (E), i.e. after tubeness analysis, to compute medial lines of DSY2-labeled axes. (B) A representative raw image of DSY2 signal in maximal projection. Scale bar represents 5 μm.(C) A pre-processed image of DSY2 signal in maximal projection after step (a).(D) Segmented axes from a single z section generated by step (b).(E) The resulting image of a representative nucleus in maximal projection after the tubeness analysis in step (c). (F) A skeletonized image of chromosome axes in maximal projection. (TIF) [file pgen.1007881.s014.tif]

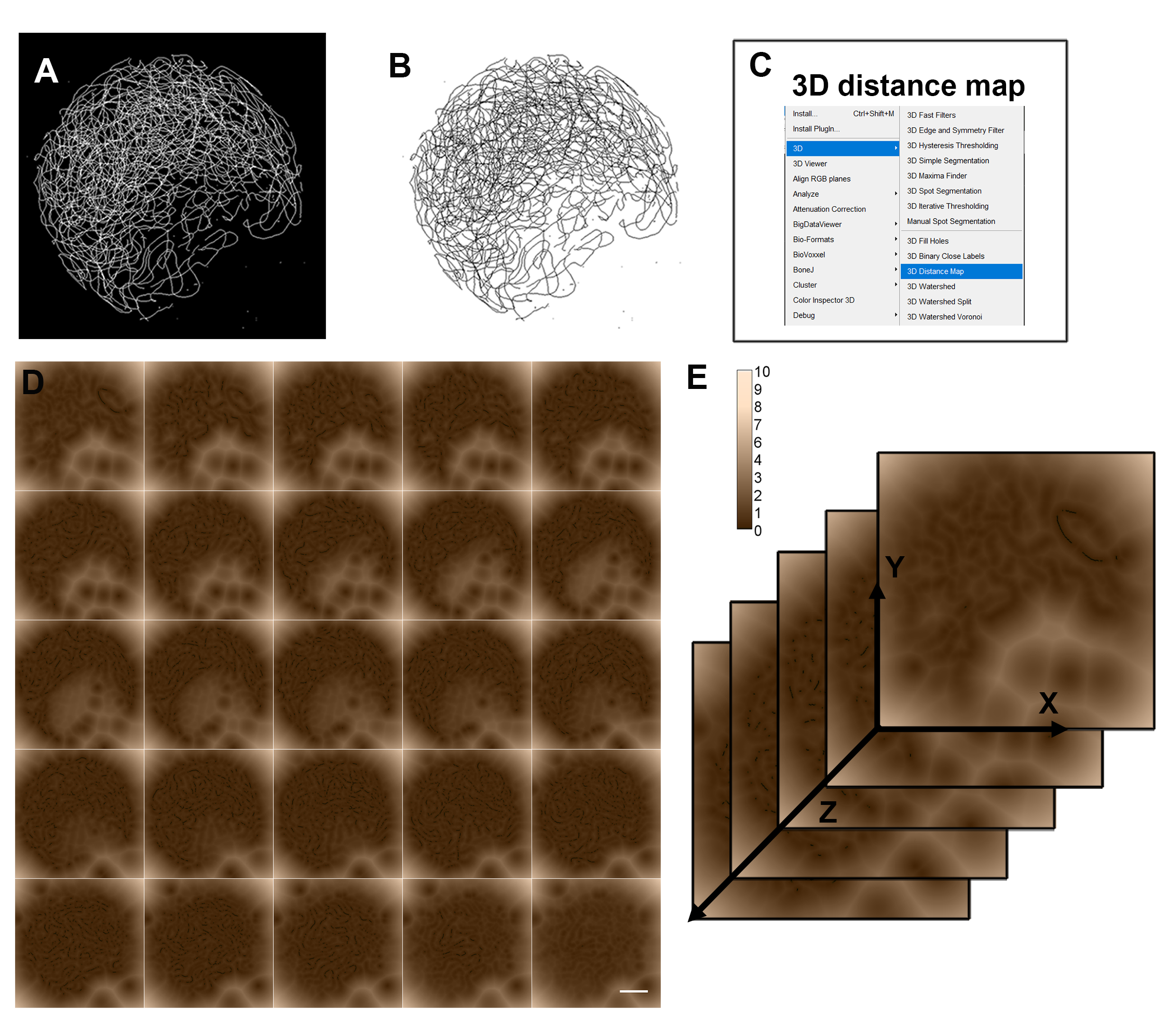

Supplement: S15 Fig — (A) A skeletonized image of chromosome axes in maximal projection.(B) The same image (A) converted to white background.(C) Screenshot of the 3D distance map tool in ImageJ.(D, E) A 3D distance map was generated from the skeletonized axis model (B) using the 3D distance map tool. These maps could be visualized in 2D by using color to denote the distance from a given point (i.e. medial lines of the skeletonized axis) to neighboring voxels. A montage of serial z sections (D) and a schematic image of Z stacks (E) of the resulting 3D distance map are shown. The color scale represents assigned distances (μm). (TIF) [file pgen.1007881.s015.tif]

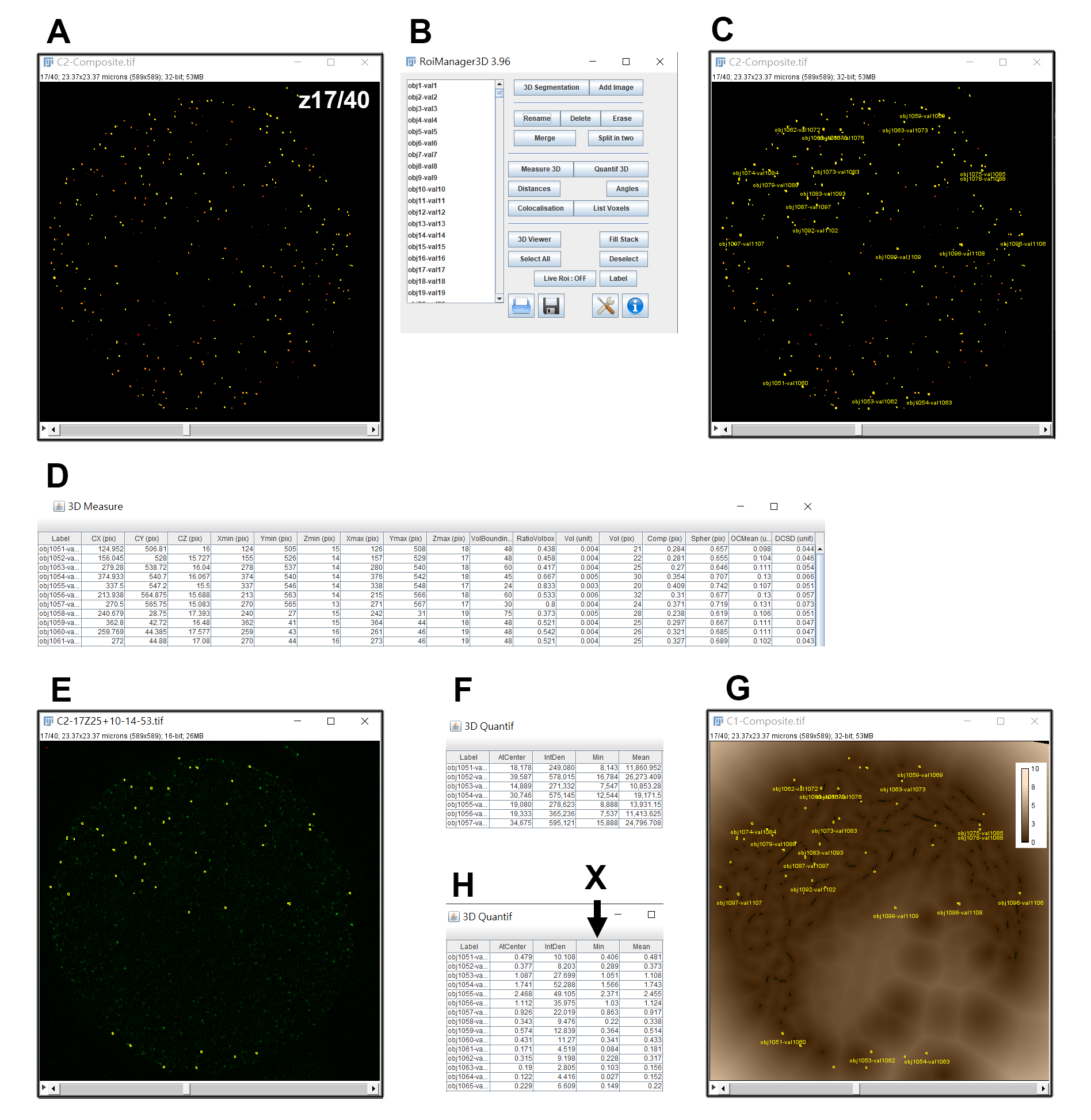

Supplement: S16 Fig — (A) A representative image of segmented SPO11-1 signals shown in a single Z section. The segmentation method for the same cell is demonstrated in S12 Fig. (B) The 3D ROI manager tool is opened and the segmented binary image is imported to add all labeled objects.(C) Examination of selected objects in the segmented binary image using 3D ROI manager.(D) Acquisition of geometric measurements of selected objects, including coordinates of the center of objects (CX, CY, CZ), the borders of objects, their volumes (Vol) and compactness (Comp), as well as the distances from the centers to borders of objects (DC). (E) Examination of selected objects (circled in yellow) from the original gray-scale image. (F) Acquisition of the fluorescence intensity of selected objects from the original gray-scale image. Signal intensity values for the center point (AtCenter), total sum (IntDen), minimal (Min) and the mean (Mean) for the objects are obtained. (G) After incorporating the 3D distance map into the 3D ROI manager, selected objects (circled in yellow) could be examined on the 3D distance map. The segmentation and skeletonization method for chromosome axes of the same cell is demonstrated in S14 Fig.(H) Acquisition of distances (μm) between the centers of selected objects to the closest axes (AtCenter), as well as minimal (Min) and mean (Mean) distances from the borders of objects to the closest axes. Axis-associated SPO11-1 objects are defined when X values (i.e. the minimal distance from AEs to borders of an object) equal zero. (TIF) [file pgen.1007881.s016.tif]

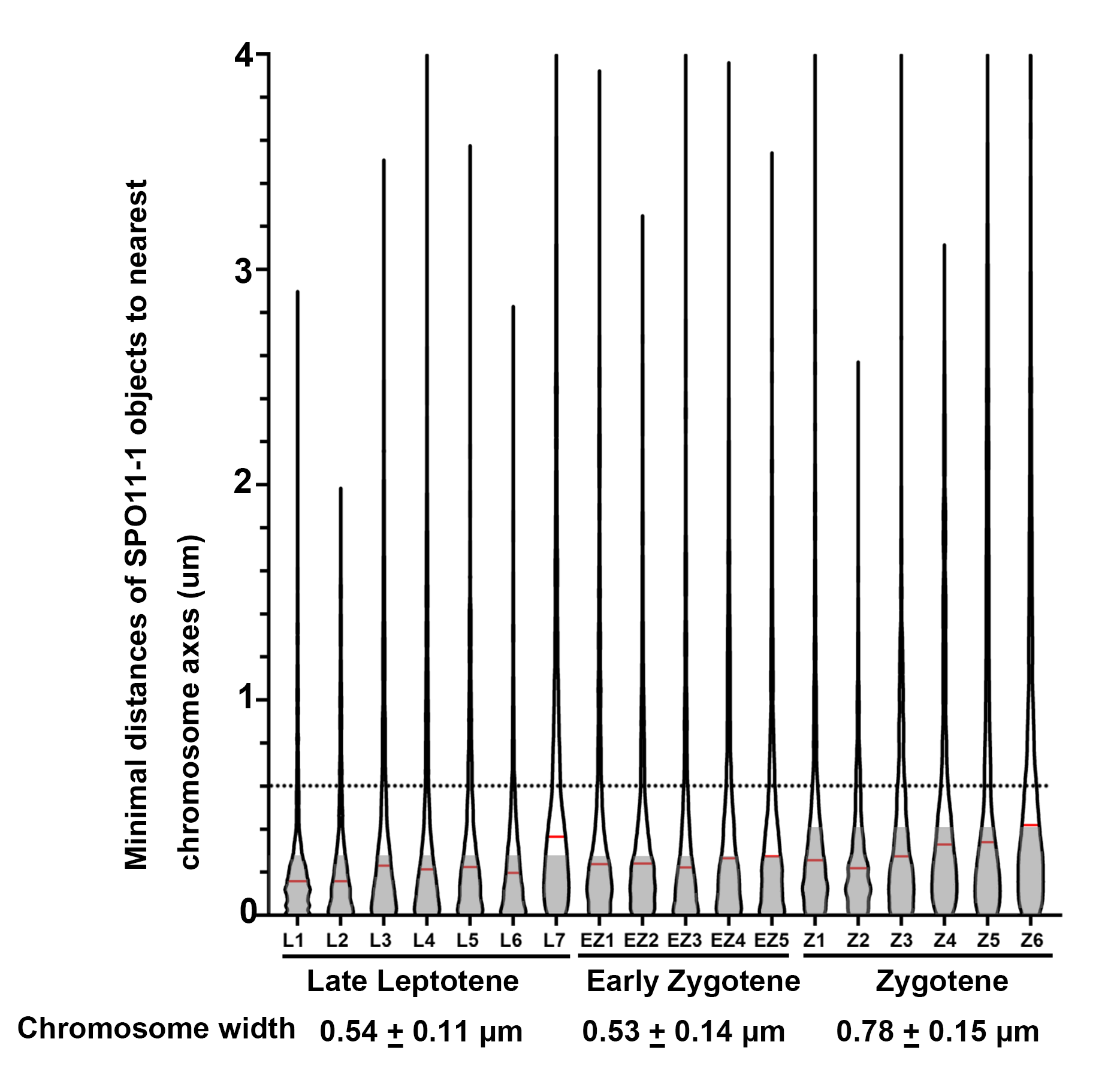

Supplement: S17 Fig — A total of eighteen meiocytes was analyzed, including seven late leptotene (L), five early zygotene (EZ) and six zygotene (Z) cells. Median values are indicated by red lines. The dotted line represents a radius of 0.6 μm from an axis. The shaded area represents estimated DAPI-stained chromosomal regions away from the axes (i.e. half the value of chromosome width). Chromosome widths were estimated by measuring at least 100 positions. (TIF) [file pgen.1007881.s017.tif]

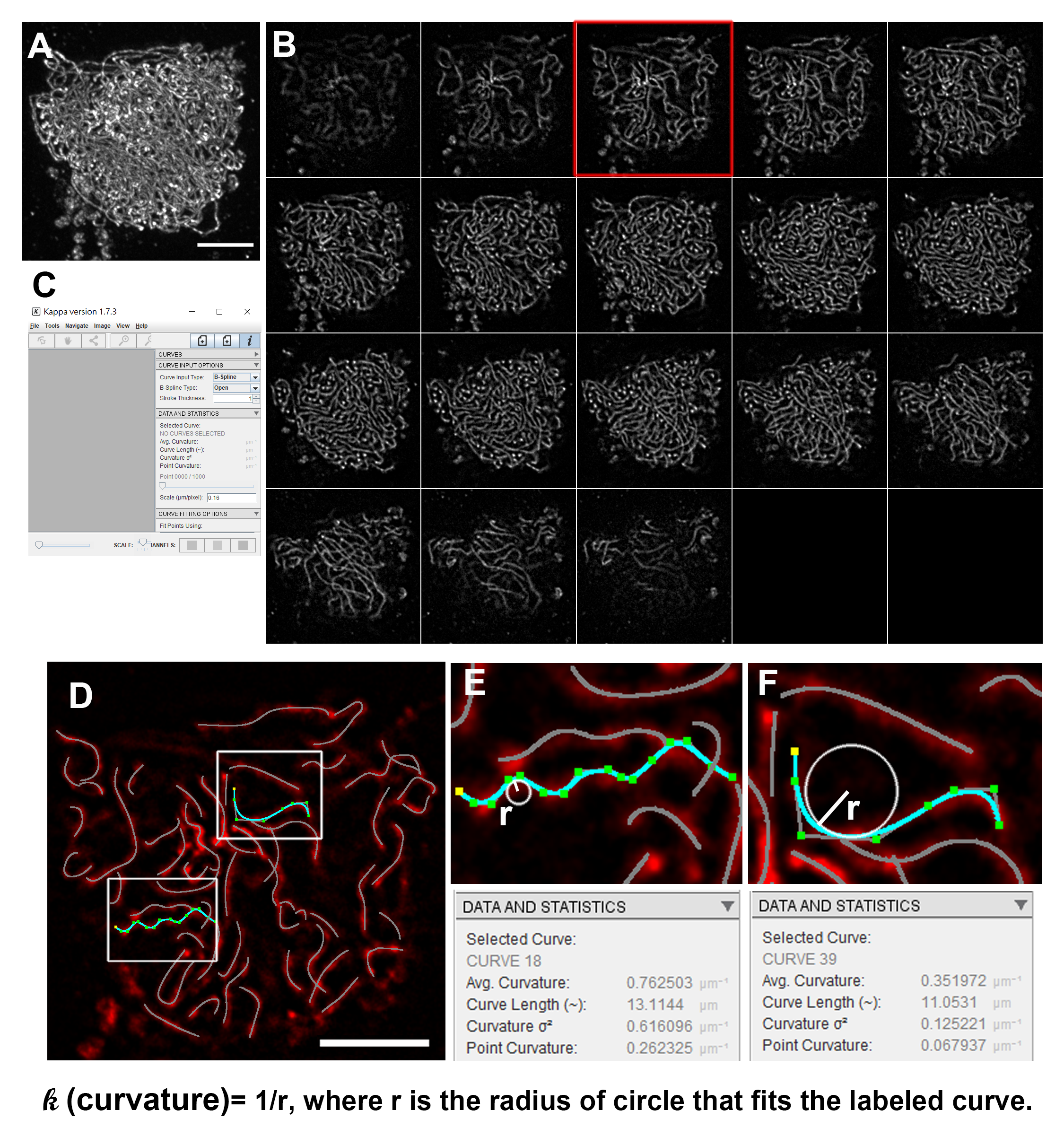

Supplement: S19 Fig — (A) Immunostaining image of DSY2 signal in maximal projection. Scale bar represents 5 μm.(B) A montage of single Z sections. (C) The Kappa plugin in ImageJ. (D) An example of one Z section (indicated by the red box in B) subjected to Kappa analysis. DSY2 stretches are traced and marked by blue lines. (E and F) Magnified regions in D. Measurements of the labeled curves are shown below the screenshots. The Kappa value of the curve is calculated by 1/r, where r is the radius of a circle that fits the points along the curve. A bigger Kappa represents a curve bend more sharply. As shown here, average kappa values of curve 18 (E) and curve 39 (F) are 0.762503 and 0.351972 μm-1, respectively. (TIF) [file pgen.1007881.s019.tif]

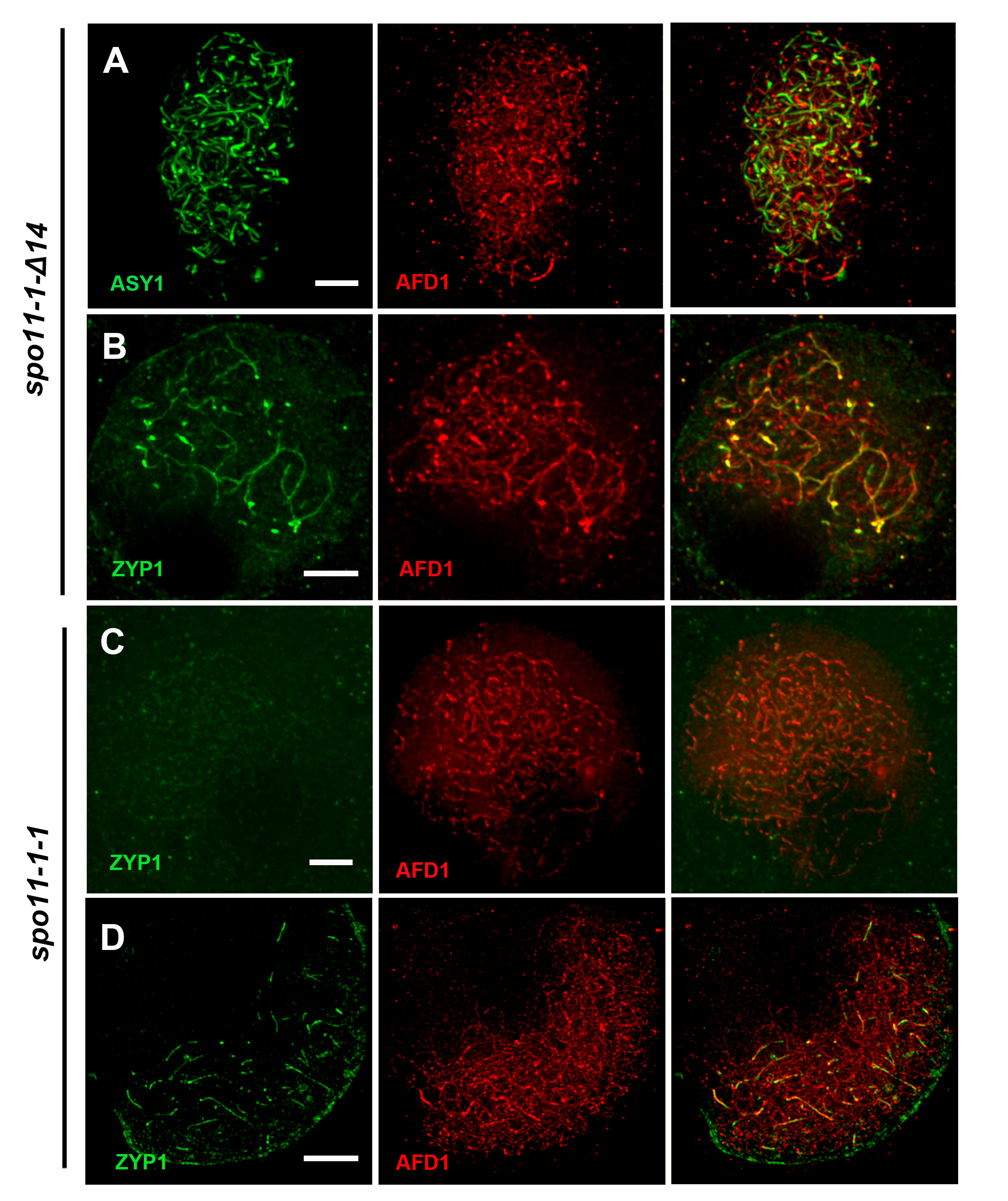

Supplement: S20 Fig — (A, B) Detection of cohesion protein AFD1/REC8, axial element ASY1 and transverse filament ZYP1 proteins at early-zygotene (A) and late-zygotene (B) of spo11-1- Δ14 mutant meiocytes showing aberrant AEs (A) and impaired SC formation (B).(C, D) Detection of cohesion protein AFD1/REC8 and transverse filament ZYP1 proteins at zygotene (C) and late-zygotene (D) in spo11-1-1 mutant meiocytes showing impaired SC formation. Scale bar represents 5 μm. (TIF) [file pgen.1007881.s020.tif]

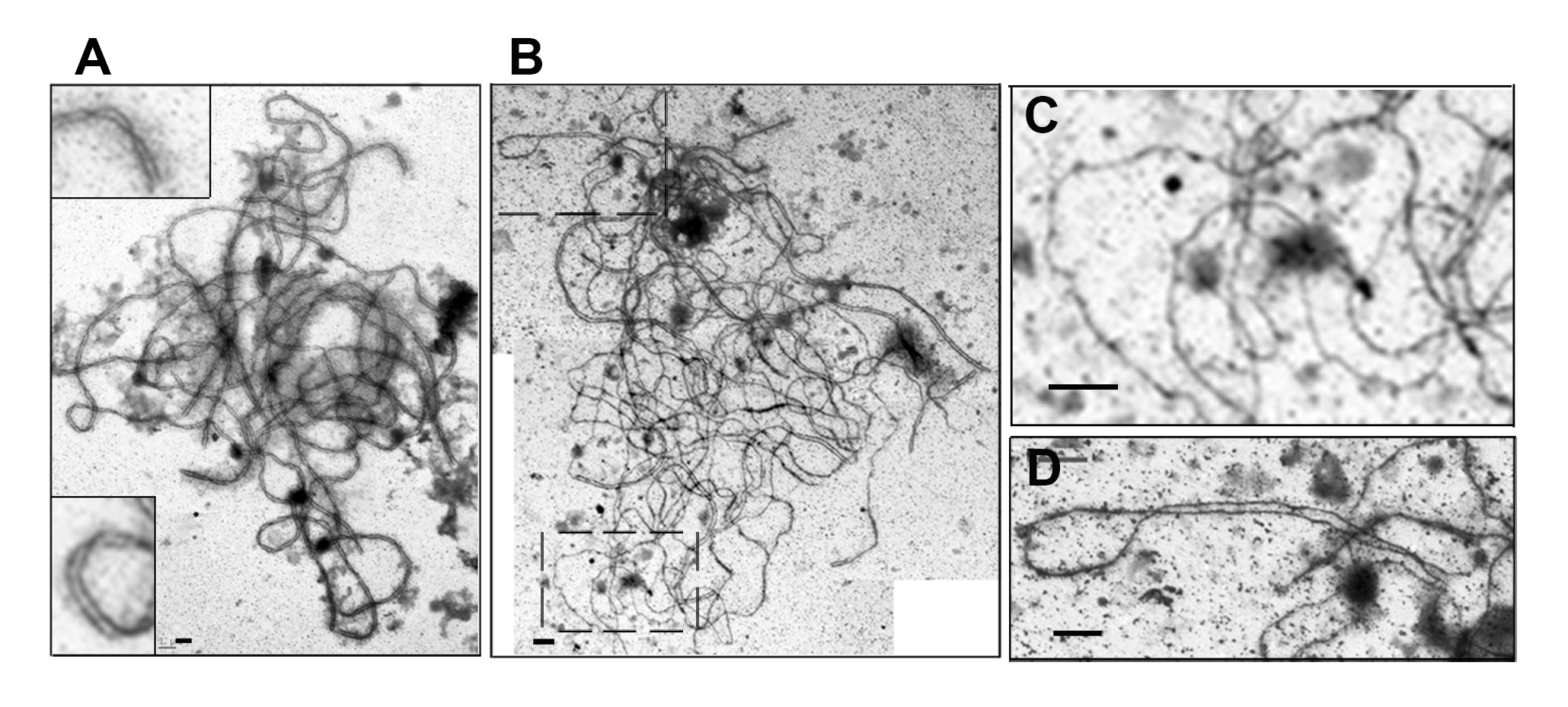

Supplement: S21 Fig — (A) WT pachytene cell exhibiting complete synapsis. (B) Promiscuous synapsis in the spo11-1- Δ14 mutant. A stitched image is shown here due to the oversized spread of the synaptonemal complex of maize meiocytes. (C, D) Higher magnifications from (B). Abnormally curly AE in unsynapsed regions (C). Abnormal fold-back synapsis (D). Scale bars represent 1 μm. (TIF) [file pgen.1007881.s021.tif]
